# Imposed work of breathing of 16 neonatal CPAP-devices using different mechanisms of CPAP generation

**DOI:** 10.1038/s41390-025-04265-w

**Published:** 2025-07-25

**Authors:** Hanna Sterzik, Joerg Arand, Christoph E. Schwarz, Matthias Kumpf, Martin Wald, Angela Kribs, Wanda Lauth, Maximilian Gross, Christian F. Poets, Bianca Haase

**Affiliations:** 1 Department of Neonatology, University Children’s Hospital, Tübingen, Germany; 2 Department of Neonatology, Center for Pediatric and Adolescent Medicine, University of Heidelberg, Heidelberg, Germany; 3 Department of Pediatric Cardiology, Pulmonology and Intensive Care Medicine, University Children’s Hospital, Tübingen, Germany; 4 Division of Neonatology, Department of Pediatrics and Adolescent Medicine, Paracelsus Medical University Salzburg, Salzburg, Austria; 5 Department of Neonatology, Children’s and Adolescents’ Hospital, University Hospital of Cologne, Faculty of Medicine, University of Cologne, Köln, Germany; 6 Team Biostatistics and Big Medical Data, IDA Lab Salzburg, Paracelsus Medical University Salzburg, Salzburg, Austria; 7 Research Programme Biomedical Data Science, Paracelsus Medical University Salzburg, Salzburg, Austria; 8 Department of Pediatrics, District Hospital Reutlingen, Reutlingen, Germany

## Abstract

**Background and Objectives:**

Failure of continuous positive airway pressure (CPAP) in preterm infants may result from respiratory exhaustion, potentially also evoked by the CPAP device’s imposed work of breathing (iWOB). This study investigated in-vitro the iWOB of 16 CPAP-devices in four simulated neonatal scenarios with focus on the impact of different mechanisms of CPAP-generation.

**Methods:**

A neonatal lung model and 3D-printed nasopharyngeal dummies (representing a 1000 g preterm and a 3000 g term infant, settings: preterm/term: V_tid_ 4.5/14 ml, rate 80/60 bpm) were used to evaluate common CPAP-devices under various conditions (two levels of positive end-expiratory pressure: 5, 10 cmH_2_O).

**Results:**

During 9300 simulated breaths, iWOB differed widely across investigated devices. The lowest inspiratory iWOB were observed in Bubble CPAP, variable-flow devices and constant-flow devices with turbine (0.04/0.15–0.17 mJ/breath for preterm/term model resp. 0.07/0.24–0.33 mJ/breath). Devices with mechanical regulation and with constant resistance require higher inspiratory effort and are therefore not relevant in the low iWOB group. The highest iWOB (0.41/2.92 mJ/breath at 5 cmH_2_O) was found in a T-piece device. The mechanism of CPAP generation influenced iWOB levels.

**Conclusions:**

The magnitude of iWOB varied considerably between devices. This should be considered, to optimize non-invasive respiratory support in neonates.

**Impact:**

- This bench study detected differences in the imposed work of breathing (iWOB) between 16 different CPAP devices.
- In addition to existing literature, the present study contributes to a more comprehensive understanding of iWOB and the influence of CPAP generation due to the large number of CPAP devices investigated.
- If the identified differences are confirmed in clinical practice, this could streamline decision-making in CPAP device selection.

## Introduction

Over the last decades, non-invasive respiratory support, including continuous positive airway pressure (CPAP), is increasingly used during resuscitation and stabilization of premature infants^1^ as it can often prevent the use of mechanical ventilation.^2^ CPAP failure, however, leads to mechanical ventilation^3^ and intubation,^4^ associated with increased risk of mortality and morbidity, including bronchopulmonary dysplasia (BPD).^5^ A reduced imposed work of breathing (iWOB) is considered an important contributor to reducing the incidence of respiratory failure^4^ possibly by reducing the respiratory effort and avoiding exhaustion. For optimizing CPAP application,^6^ the evaluation of iWOB, particularly inspiratory iWOB (iWOB_insp_), should be further pursued, as this could impact short- and long-term outcome of term and preterm infants.

As the pressure stability of the device depends on the mechanism of CPAP-generation (jet-flow devices vs. constant-flow devices), the latter may influence iWOB.^1,7–9^ Jet-flow devices demonstrated a positive effect on iWOB,^9–11^ expiratory resistance,^12^ oxygen demand,^13,14^ along with higher success rates after extubation^15^ and improved lung recruitment^6,9^ compared to continuous flow devices. Additionally, CPAP-devices differ in their technological complexity and therefore require different resources for set-up, maintenance and repair, affecting their use in resource-limited countries. However, not all CPAP devices offer mechanical ventilation, which may become necessary if CPAP fails.

The aim of this study was to assess iWOB_insp_ across commonly used neonatal CPAP-devices through an in-vitro comparison, to guide clinical practice.

## Methods

### Experimental setup and examined CPAP-devices

In this experimental study in- and expiratory pressure fluctuations were measured using the neonatal active lung model (NALM®; Dr. Schaller Medizintechnik, Dresden, Germany). The NALM was calibrated before starting measurements. All respiratory parameters were configured to mimic term (3000 g) and preterm (1000 g) infants (online Supplementary, Table S1). Two nasopharyngeal space-dummies based on three-dimensional data^16^ and commonly used prong sizes were constructed by Printoptix Inc. (Stuttgart, Germany) and realized using 3D-printing (online Supplementary, Fig. S1, STEP files can be provided upon request).

The NALM’s driver stream was connected to the device’s interface, subsequently linked to the respective face and nares dummy through device- and patient-specific prongs for each of 16 investigated CPAP-devices (Table 1). For devices with CPAP display, CPAP was set according to the display on the device. For devices without a display [Benveniste^®^, NeoBreathe^®^, Perivent^®^ (/Neopuff^®^, T-piece device)], CPAP was set using the P_Y_ (pressure on the y-piece) graph on the Graphical User Interface (GUI). Acquired measurements were transferred via USB to a laptop computer (online Supplementary, Fig. S1), where they were converted to a tdms data file using the GUI and thus converted into .xlsx using the Excel add-in from National Instruments (TDM-Importer Version 21.3.0 49496; National Instruments, Austin-Texas, USA). The Excel file was employed to calculate iWOB and conduct statistical analyses through custom scripts in Matlab (MATLAB R2022b, Natick, MA, the scripts can be provided upon request).

**Table 1:** Summary of the 16 investigated CPAP devices and interfaces.

CPAP-devices		
Technical principle	CPAP-Device	Can be used for mechanical ventilation
***CPAP-device with variable flow (Jet-flow)***		
	Original Benveniste valve® (Dameca, Kopenhagen, Denmark)***	no
	NeoBreathe valve® ^a^ (PFM, Koeln, Germany)***, more information in the online supplement	no
	Infant flow® (Viasys Healthcare, Conshohocken, USA)*	no
	RPAP® (Inspiration Healthcare, Crawley, UK)**	yes
	Medijet® (Medin Medical Innovations, Munich, Germany)**	no
***CPAP-device with constant flow***		
electrically regulated via Turbine	Eve® (Fritz Stephan GmbH Medizintechnik, Gackenbach, Germany)***	yes
electrically regulated	Leoni + ® (Löwenstein, Bad Ems, Germany)***	yes
electrically regulated	Leoni4®^ a^ (Löwenstein)***	yes
electrically regulated	Sophie® (Fritz Stephan)***	yes
electrically regulated	Babylog 8000plus® (Dräger, Luebeck, Germany)***	yes
electrically regulated via Turbine	Hamilton-T1 ® (Hamilton Medical, Bonaduz, Switzerland)***	yes
electrically regulated	Servo-i ® (Maquet, Rastatt, Germany)***	yes
electrically regulated	Servo n® (Maquet)***	yes
Mechanically regulation (spring valve)	F120® (Fritz Stephan)***	yes
constant resistance	Perivent® (t-piece device) (Fisher and Paykel Healthcare, Auckland, New Zealand)***	yes
***Bubble-CPAP***		
Bubble-CPAP	Bubble CPAP® (Dräger)***	no
Interfaces		
**Interface**	**Preterm Model**	**Term model**
Prong Infant Flow (Conshohocken, Pennsylvania, USA)*	size S	size L
	ID: 3.6 mm	ID: 4.0 mm
	OD: 4.2 mm	OD: 5.0 mm
Prong RPAP (Mettawa, USA)**	size M	size L
	ID: 3.5 mm	ID: 4.0 mm
	OD: 4.5 mm	OD: 5.0 mm
Prong Stephan „EasyFlow nCPAP“ (Gackenbach, Germany)***	size M	size L
	ID: 3.1 mm	ID: 4.2 mm
	OD: 4.1 mm	OD: 5.2 mm

Symbols “* / ** / ***” indicate the interfaces utilized in the test of the 16 CPAP devices.

*ID* inner diameter, *OD* outer diameter.

^a^presently undergoing licensing for sale.

All measurements assumed that gas viscosity had no influence on iWOB.^17^ To ensure standardized conditions, all respiratory cycles were performed with non-humidified air at room temperature.

### Calculation of iWOB

The main parameter to be compared between devices was iWOB, computed as the area enclosed by the graph. As this model of NALM did not provide an automated calculation of iWOB, we calculated iWOB as the trapezoidal integration of Δp (the difference of P_y_ to CPAP) with respect to V. P_Y_ represents the pressure at the y-piece simulating pharyngeal pressure, and V denotes the volume of the piston-cylinder system simulating lung volume.

Airway pressure was determined experimentally as the mean value of P_Y_ before simulated breathing was started. P_Y_, decreasing during expiration and increasing during inspiration, was used to define and analyze ventilator breaths. As the Bubble CPAP generates oscillations originating from its air bubbles, the curve was smoothed using a moving average so that iWOB could be calculated.^18^ The resulting total iWOB (iWOB_tot_) consists of the sum of inspiratory (iWOB_insp_) and expiratory (iWOB_exp_) iWOB. Only the inspiratory iWOB_insp_ was evaluated for statistical analysis, as increased iWOB_exp_ only results in an increased expiration time.^19^

Preliminary tests have shown that plugging and unplugging the hoses can significantly influence iWOB. To distribute the influence of plugging and unplugging among as many groups as possible, 10 measurements of 30 s duration each were taken per device, model and CPAP level. For each measurement, 15 breaths were evaluated during steady state (from at least the sixth inspiration onwards). Between all 10 measurements, the ventilator was disconnected, gas flow interrupted, and respiratory settings were reset in the NALM and in the corresponding CPAP-device. Using this experimental design, 620 experiments were performed.

### Statistical analysis

For case number planning, the difference to be detected was set at 0.059 (preterm) resp. 0.2 mJ/breath (term) which reflects 5% of the physiologic work of breathing (online Supplementary Table S2). The standard deviation (SD) observed in preliminary tests was 0.031 (preterm) resp. 0.065 mJ/breath (term). As a total of more than 100 pairwise device comparison was planned, the significance level alpha was set at 0.05% (adjusted significance level of the individual tests for multiple testing according to Bonferroni). The power was set at 80%. The result of this sample size calculation was *n* = 85. As the plugging and unplugging was observed as a confounding effect on iWOB, the number of cases was increased to 150 to spread the measured values for iWOB to as many groups (*n* = 10) of plugging and unplugging as possible.

A two-factor analysis of variance (ANOVA) was performed to account for the confounding effect of plugging and unplugging. The two main factors (device and plugging/unplugging) and a possible interaction between these factors were considered. At *p* < 0.0005, a statistically significant difference was assumed, except if an interaction between the two main factors was detected. The interaction indicates that the differences in iWOB between two devices is not due to the device but due to the (un)plugging (online Supplementary, Figs. S3, S4). Subsequently, the pairwise analysis was performed using the Tukey-Kramer test as a post-hoc test. A *p* value < 0.05 was considered statistically significant. The confidence interval was defined as 95% (α = 5%).

## Results

A total of 9300 breaths (4800 in the preterm infant model) were evaluated and considerable differences in iWOB_insp_ detected between devices investigated (online Supplementary Tables S3 and S4). In general, the lowest iWOB_insp_ was detected with the Bubble CPAP^®^ (in the preterm model 0.04 mJ/breath at CPAP 5 cmH_2_O resp. 0.03 mJ/breath at 10 cmH_2_O and in the term model 0.17 mJ/breath at both CPAP levels, Fig. 2 and online Supplementary Fig. S2), followed by the Hamilton-T1^®^ (in the preterm model 0.04 mJ/breath at CPAP 5 cmH_2_O resp. 0.06 mJ/breath at 10 cmH_2_O and in the term model 0.17 mJ/breath at CPAP 5 cmH_2_O resp. 0.27 mJ/breath at CPAP 10 cmH_2_O). Benveniste^®^ and NeoBreathe^®^ as variable-flow devices and Eve^®^ as continuous flow device showed the third lowest iWOB_insp_ [in the preterm model at CPAP 5 and 10 cmH_2_O: ~ 0.07 mJ/breath, in the term model at this pressure: ~ 0.02–0.03 mJ/breath (both <5% of WOB_phys_)]. As multiple interactions were observed among these five devices (online Supplementary Figs. S3, S4), detected differences cannot reliably be interpreted as statistically significant. This is because the differences might also result from the process of (un)plugging, rather than representing true device differences.

The RPAP^®^, although the only variable-flow-device allowing for mandatory ventilation breaths and causing the lowest iWOB_insp_ in the preterm model [at CPAP-level of 5 cmH_2_O (0.05 mJ/breath), online Supplementary Table S3A], showed a wide variation between measured iWOB (e.g. standard deviation (SD) up to 0.029) and high values for iWOB_insp_ in the term model (0.12mJ/breath at CPAP 5 cmH_2_O, respectively 0.84 mJ/breath at CPAP 10 cmH_2_O, online Supplementary Table S4A&B).

With an iWOB_insp_ of about 0.07mJ/breath, similarly low values were measured with Benveniste^®^, NeoBreathe^®^ and InfantFlow^®^ in the preterm model at CPAP 5cmH_2_O. In all other simulated scenarios, the use of Benveniste^®^, NeoBreathe^®^ and Eve^®^ resulted in lower iWOB_insp_ (online Supplementary Tables S3B and S4) than seen with the other CPAP-devices (*p* < 0.05, Fig. 2).

**Fig. 1:**
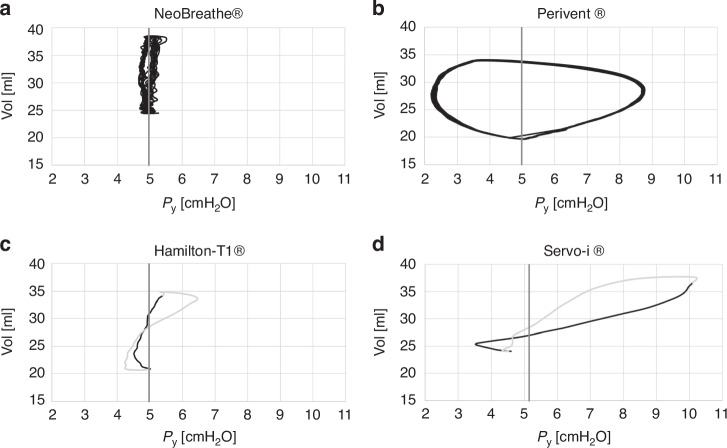
Pressure [cmH_2_O] - volume [ml] (p-V) loops for different CPAP devices for the term model and CPAP 5 cmH_2_O (exact measured CPAP displayed by vertical line). Panels **a** and **b** multiple breaths; **a** NeoBreathe®: example for low iWOB, **b** Perivent® (T-piece device): example for high iWOB; **c** and **d** one exemplary breath, dark part of the loop: inspiration, light part of the loop: expiration; **c** Hamilton-T1® showing loop-crossing, **d** Servo-i® showing loop-crossing possibly inspiratory strokes triggered by the simulated breaths.

The Perivent^®^ was constantly identified as having the highest iWOB_insp_, which could be almost twentyfold the iWOB_insp_ found for the device with the lowest measured iWOB_insp_ (see exemplary NeoBreathe^®^, Fig. 1a).

The Servo-i^®^ showed a negative iWOB_insp_. Furthermore, since the p-V-loop was atypical for CPAP devices (Fig. 1d) and the device had a high maximum pressure, it was not included in the statistical analysis.

**Fig. 2:**
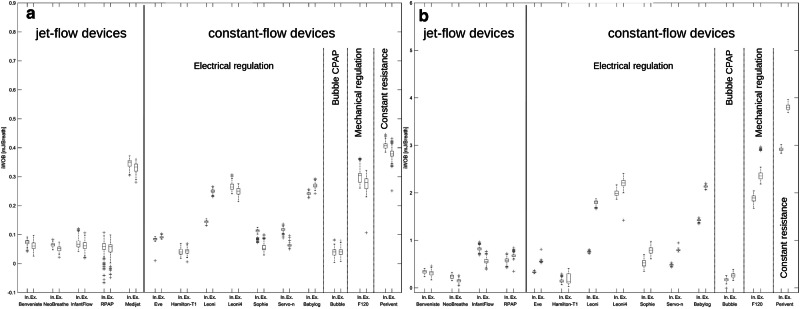
Box-plots of proportion of additional iWOB_insp_ (CPAP 5 cmH_2_O). Inspiratory iWOB in a simulated 1000 g preterm (**a**) resp. 3000 g term (**b**) model (CPAP 5 cmH_2_O, see online supplement for 10 cmH_2_O and tables S3 and S4 for additional parameters); no iWOB determination feasible in term model for Medijet® and both models for Servo-i®.

An effect of CPAP generation mechanism on iWOB_insp_ was observed. Variable-flow-devices such as Benveniste^®^ and NeoBreathe^®^, as well as Bubble CPAP^®^ and most constant-flow-devices with electrical regulation and automated flow regulation (Eve^®^, Sophie^®^, Servo-n^®^) tended to result in lower iWOB_insp_ except for Leoni4^®^ (flow default 4 l / min) than passive devices with fixed flow settings (Leoni + ^®^, Babylog^®^). Constant-flow-devices with mechanical regulation (F120^®^) or constant resistance (Perivent^®^) exhibited higher values (Fig. 2 and online Supplementary, Fig. S2).

However, it should be noted that the iWOB_insp_ associated with variable-flow-devices varied considerably: while Benveniste^®^ and NeoBreathe^®^ showed very low iWOB_insp_ across all simulated scenarios, this was only the case for InfantFlow^®^ and RPAP^®^ in preterm infant simulations (Fig. 2 and online Supplementary, Fig. S2). In contrast, Medijet^®^, resulted in a comparatively high iWOB_insp_ even at preterm infant simulation.

Furthermore, a positive influence of increasing CPAP on iWOB_insp_ was detected for most constant-flow-devices tested with electronic flow regulation (Eve^®^, Sophie^®^, Leoni + ^®^, Babylog^®^), while this effect was not seen with the other devices.

## Discussion

In this in-vitro comparison, we assessed a diverse array of devices employing various technologies commonly used for non-invasive respiratory support in preterm and term infants. Our study encompassed both, high-end neonatal ventilators and simpler devices designed for generating CPAP. Notably, this is the inaugural investigation to consider the various types of CPAP generation among passive devices and their impact on iWOB_insp_, while also examining the reciprocal effect of increasing CPAP levels in passive devices with electronic flow regulation. Moreover, our thorough evaluation, analyzing 9300 breaths conducted under standardized conditions, facilitated a reliable comparison of various CPAP devices used in neonatology.

As also true in clinical practice, improvement in respiratory mechanics could be seen with every device investigated. It seems that the benefit from this respiratory support is on another scale than the additional respiratory effort due to iWOB_insp_. Nonetheless, our findings unveiled discrepancies among CPAP devices regarding iWOB_insp_ which could compromise the respiratory benefit induced by the CPAP device. This could lead to differences in efficacy between devices.

Bubble CPAP^®^, still widely used in many countries, showed the lowest iWOB_insp_. To estimate iWOB_insp_ despite the oscillations caused by the CPAP generator, we had to smooth the curve using a moving average. This shaped the pressure-volume loop into a narrower loop around the CPAP pressure and balanced out the fluctuations in the generated CPAP. Furthermore, some loops (especially those during the simulation of the preterm infant) showed loop crossing. This hindered the calculation of iWOB and may have led to an unrealistically low iWOB value. Nonetheless, it should be noted that Bubble CPAP^®^ could improve gas exchange in-vivo due to the oscillations which can be understood as “light HFO (high frequency oscillation)”, and thus further minimize the work of breathing.^20^

Moreover, the p-V-Loop of Hamilton-T1^®^ also had a loop crossing. This may be due to a potential overcompensation at the end of each breath, which could explain the low iWOB_insp_. At the same time, however, the exploitation of this mechanism could also minimize comparability with the other devices investigated. Benveniste^®^, NeoBreathe^®^, and Eve^®^ emerged as having the second lowest iWOB_insp_, demonstrating less susceptibility to changes in tidal volume or respiratory rate compared to InfantFlow^®^, RPAP^®^, and Perivent^®^. As most of these pressure-volume loops did not contain any loop crossings and the curves had not been smoothed, the calculated results for iWOB probably reflect reality reliably. Notably, Perivent® consistently yielded significantly higher iWOB_insp_, reaching up to 120% of the physiological work of breathing (Fig. 2, online Supplementary Table S2). Despite RPAP^®^ showcasing low iWOB_insp_ under simulated preterm infant conditions at 5cmH_2_O with the potential for inspiratory breaths, its performance was deemed inferior to Benveniste^®^, NeoBreathe^®^, and Eve^®^ due to high standard deviations and substantial increases in iWOB_insp_ during variations in tidal volume or respiratory rate. As this has not yet been reported, further research is needed to investigate the pressure stability of this device. The observed increase in iWOB_insp_ with InfantFlow^®^ may be due to the smaller difference between system flow and maximum inspiratory flow, highlighting the limitations of this type of CPAP in larger patients. Nonetheless, the precise mechanism underlying the rise in iWOB_insp_ with this device remains elusive. As this has not yet been reported, further investigations concerning the functioning of this device are needed.

The observed decline in iWOB_insp_ with increasing CPAP in most passive devices with electronic flow regulation can be ascribed to the diminishing influence of the patient’s breathing on the applied pressure level.

Servo-i^®^ showed negative values for iWOB_insp_, an atypical p-v-loop and high maximum pressures. This device could possibly have been working unnoticed in NIV mode instead of CPAP mode. If this is confirmed in-vivo, it may carry potential risks, as the detected P_Y_ could decrease by nearly 20 cmH_2_O (online Supplementary Tables S3, S4).

Although our results may not directly correlate with existing studies due to heterogeneity in study concepts, our findings align with some previous research,^4,21,22^ while discrepancies were noted to other studies,^23,24^ likely attributable to variations in methodology and tidal volumes chosen. Despite these discrepancies, the significance of iWOB_insp_ differences underscores its potential impact on CPAP success and patient outcomes.

Donaldsson et al.^4^ compared RPAP^®^ with Neopuff^®^ and another T-piece device in-vitro using a mechanical lung model. They reported total iWOB values of 0.66 mJ/breath for RPAP^®^ resp. 4.94 mJ/breath for Neopuff^®^ at 4 cmH_2_O with a tidal volume of 16 ml. Discrepancies with our findings (RPAP^®^ 1.26 mJ/breath resp. Neopuff^®^/Perivent^®^ 6.72 mJ/breath at 5 cmH2O with a simulated term infant), may stem from differences in lung models or interface design.

Drevhammar et al.^21^ assessed Bubble^®^, Benveniste^®^, Hamilton^®^, InfantFlow^®^ and Medijet^®^ using the same mechanical lung model as Donaldsson et al.^4^ Their reported total iWOB values at 4 cmH_2_O for a simulated 3.6 kg infant were: Bubble^®^: 3.68 mJ/breath, Benveniste^®^: 5.52 mJ/breath, Hamilton^®^: 2.40 mJ/breath, InfantFlow^®^: 1.49 mJ/breath and Medijet^®^: 9,48 mJ/breath. While Drevhammar et al.^21^ found lower iWOB for InfantFlow^®^ compared to Benveniste^®^, our results showed the opposite, potentially due to differences in interface design.

Kuypers et al.^22^ compared pressure stability between RPAP^®^ with Neopuff^®^ using a custom simulation model. During inspiration at 8 cmH_2_O pressure increased by 1.1 cmH_2_O with RPAP® and 5.7 cmH_2_O with NeoPuff^®^, though tidal volume or respiratory rate were not reported. Our investigations revealed deviations of 0.79 cmH_2_O for RPAP^®^ and 3.94 cmH_2_O for the Perivent^®^ from a target CPAP of 5 cmH_2_O (simulated term infant), confirming that CPAP deviation was approximately five times higher with Neopuff^®^ than with RPAP^®^. For instance, our findings regarding Perivent^®^ causing significantly higher iWOB_insp_ than all other devices investigated are consistent with those of Donaldsson et al.^4^ and Falk et al.^24^

A notable strength of our study is not only its high number of evaluated breaths but also that it accounted for factors such as plugging and unplugging in the context of a two-factor ANOVA, ensuring high reliability and repeatability compared to other studies. Since we only simulated two patients, a healthy preterm and a respiratory ill term, the transferability to the entire patient clientele seen in neonatology is limited. Future research involving a broader range of simulated patient models are needed to generalize the findings to most neonatal patients. Additionally, it should be noted that, despite minimizing leaks as much as possible - by ensuring a closed mouth and nostrils adapted to prong size - discrepancies between in- and expiratory volumes may still indicate the presence of a leak, the source of which remains unclear. Another limitation lies in the in-vitro nature, which includes a limited selection of patient scenarios. Future research, especially clinical studies, is warranted to confirm the clinical relevance of our findings.

Nonetheless, given that the observed iWOB_insp_ ranges from approximately 5% to 120% of the physiological work of breathing (Fig. 2, online Supplementary Table S2), it seems reasonable to assume that iWOB_insp_ exerts a potentially influence on CPAP therapy success and clinical outcomes in some scenarios.

## Conclusion

Variability in iWOB_insp_ between different devices and technologies used for CPAP treatment exists. The lowest iWOBinsp was found with Bubble CPAP^®^, Hamilton-T1^®^, Benveniste^®^, NeoBreathe^®^ and Eve^®^. If these results are confirmed in vivo, the choice of a CPAP device with low iWOB could potentially improve the clinical outcome in individual cases.

## Supplementary information

Online Data Supplemental

## Data Availability

The datasets generated during and/or analysed during the current study are available from the corresponding author on reasonable request.
