# Correction: Imposed work of breathing of 16 neonatal CPAP-devices using different mechanisms of CPAP generation

**DOI:** 10.1038/s41390-025-04640-7

**Published:** 2025-12-03

**Authors:** Hanna Sterzik, Joerg Arand, Christoph E. Schwarz, Matthias Kumpf, Martin Wald, Angela Kribs, Wanda Lauth, Maximilian Gross, Christian F. Poets, Bianca Haase

**Affiliations:** 1 Department of Neonatology, University Children’s Hospital, Tübingen, Germany; 2 Department of Neonatology, Center for Pediatric and Adolescent Medicine, University of Heidelberg, Heidelberg, Germany; 3 Department of Pediatric Cardiology, Pulmonology and Intensive Care Medicine, University Children’s Hospital, Tübingen, Germany; 4 Division of Neonatology, Department of Pediatrics and Adolescent Medicine, Paracelsus Medical University Salzburg, Salzburg, Austria; 5 Department of Neonatology, Children’s and Adolescents’ Hospital, University Hospital of Cologne, Faculty of Medicine, University of Cologne, Köln, Germany; 6 Team Biostatistics and Big Medical Data, IDA Lab Salzburg, Paracelsus Medical University Salzburg, Salzburg, Austria; 7 Research Programme Biomedical Data Science, Paracelsus Medical University Salzburg, Salzburg, Austria; 8 Department of Pediatrics, District Hospital Reutlingen, Reutlingen, Germany

Correction to: *Pediatric Research* 10.1038/s41390-025-04265-w, published online 25 July 2025

In this article the figure for online supplemental with CPAP 10 cmH2O was used instead of the correct Fig. 2.; the figure should have appeared as shown below.

Former Fig 2:
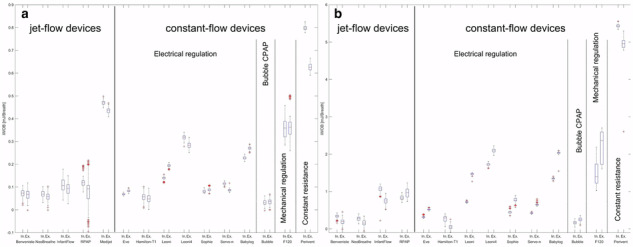


Correct Fig 2:
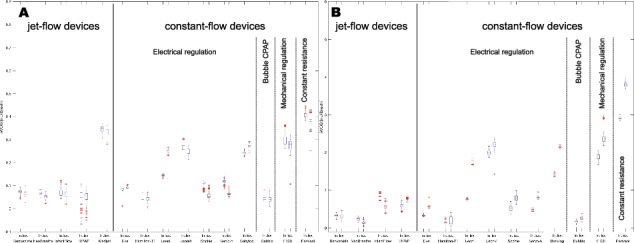


The original article has been corrected.

