# Supplementary material for: Imposed work of breathing of 16 neonatal CPAP-devices using different mechanisms of CPAP generation
Source: Pediatr Res. 2025 Jul 25;99(3):1187–92. doi: 10.1038/s41390-025-04265-w (PMC13021497; doi:10.1038/s41390-025-04265-w)
Supplement: Supplementary file 1 — Online Data Supplemental [file 41390_2025_4265_MOESM1_ESM.pdf]

Online Data Supplement

Imposed work of breathing of 16 neonatal CPAP-devices using different mechanisms of CPAP generation

Hanna Sterzik[1], Joerg Arand[1], Christoph E. Schwarz[1, 3*], Matthias Kumpf[4], Martin Wald[5], Angela Kribs[2], Wanda Lauth[6, 7], Maximilian Gross[1, 4], Christian F. Poets[1], Bianca Haase[1, 8]

[1] Department of Neonatology, University Children's Hospital of Tuebingen, Germany

[2] Department of Neonatology, University Children's Hospital of Koeln, Germany

[3] Department of Neonatology, University Hospital for Pediatrics and Adolescent Medicine Heidelberg, Germany

[4] Department of Pediatric Cardiology, Pulmonology and Intensive Care Medicine, University Children's Hospital, Tübingen, Germany

[5] Division of Neonatology, Department of Pediatrics and Adolescent Medicine, Paracelsus Medical University Salzburg, Austria

[6] Team Biostatistics and Big Medical Data, IDA Lab Salzburg, PMU Salzburg, Strubergasse 16, 5020 Salzburg, Austria

[7] Research Programme Biomedical Data Science, PMU Salzburg, Strubergasse 16, 5020 Salzburg, Austria

[8] Department of Pediatrics, District Hospital Reutlingen, Reutlingen, Germany

Table S1: Overview of the set-up, predefined respiratory parameters, and used interfaces in simulated patient models

	**preterm model, 1000g**	**term model, 3000g**
Parameter		
t_{ins} [ms]	300	400
f [/min]	80	60
Aimed V_{tid} [ml] / [ml/kg]	4.5 / 4.5	14 / 4.67
Prm [hPa]	Adjusted to ensure the target tidal volume is reached as precisely as possible	
Compliance set on the NALM	Cint	Cint
Compliance [ml / hPa] set on the GUI	1.0	1.0
Tube [mm]	3.5	3.5
Resistance	Ra1	Ra1
Defined physiologic WOB based on data [mJ / breath] (see table S2)	1.18	4.00

f: frequency, Vtid: tidal volume, tins: inspiration time

Table S2: Literature data on physiologic work of breathing (WOB)

Infant category	Publication	WOB	Study collective
Preterm	Abbasi and Bhutani 1990[S1]	12.0 g / cm / kg = 1.18 mJ / breath	healthy preterm infants with a gestational age of 28 to 34 weeks and a birth weight of < 1500 g
Newborn	Cook et al 1955 [S2]	1450 g*cm / min = 4.18 mJ / breath	Healthy preterm and term newborns (gestational age of 36 to 42 weeks)
	Cook et al 1957 [S3]	38 gm * cm = 3.73 mJ / breath	Healthy newborn infants
	Estol et al [S4]	8.65 W / l / kg / min ~ 3.26 mJ / breath	Most newborn infants

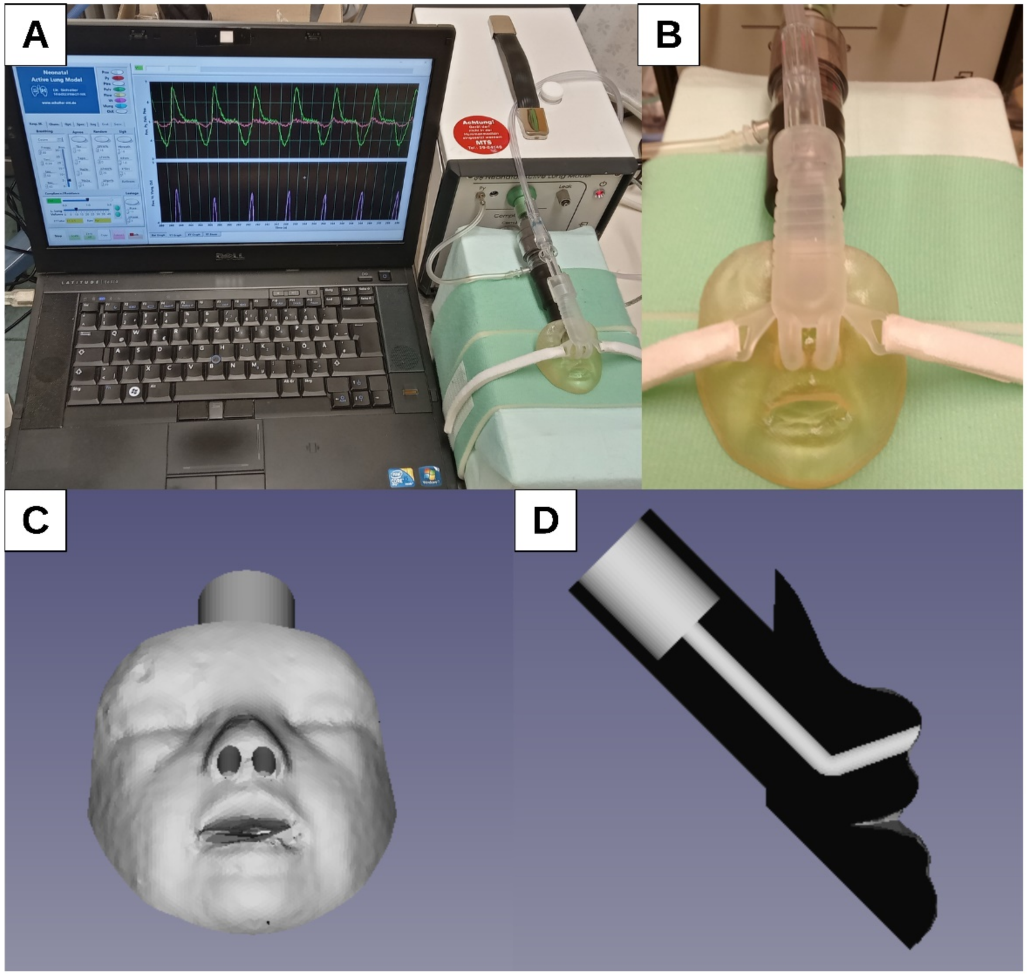

Figure S1: Details on the experimental setup, **A:** photography of the experimental setup with laptop computer, NALM, CPAP device (in the photography: NeoBreathe Valve®) and dummy, **B:** dummy and CPAP device (NeoBreathe Valve®) more detailed, **C** and **D**: 3D construction of the dummy

Information on the NeoBreathe Valve®

The NeoBreathe Valve® operates on the same principle as the Benveniste Valve®, but differs in its linear design, injection molding construction and varying diameters in both; the jet and collecting tube.

Photographs of the NeoBreathe Valve® can be seen in figure S1A and B.

Table S3: Result of the measurements CPAP 5 cmH_2O

A

CPAP-Device	P_{min} $[cmH_2O]$	P_{max} $[cmH_2O]$	$V_{tid}ex$ [ml]	$V_{tid}in$ [ml]	$iWOB_{ex}$ [mJ/breath] (SD)	$iWOB_{in}$ [mJ/breath] (SD)	$iWOB_{ges}$ [mJ/breath] (SD)	Flow [l/min]
Benveniste	4.71	5.48	4.51	4.21	0.06 (0.01)	0.07 (0.01)	0.13 (0.02)	variable
NeoBreathe	4.70	5.33	4.60	4.31	0.05 (0.01)	0.07 (0.01)	0.12 (0.01)	8
InfantFlow	4.63	5.51	4.40	4.17	0.06 (0.02)	0.07 (0.02)	0.13 (0.03)	variable
RPAP	4.65	5.48	4.56	4.29	0.05 (0.03)	0.05 (0.03)	0.10 (0.04)	12
Medijet	3.81	6.21	4.45	3.91	0.33 (0.02)	0.34 (0.02)	0.68 (0.03)	8
Eve	4.71	5.64	4.73	4.38	0.09 (0.00)	0.08 (0.01)	0.18 (0.01)	automatical
Leoni +	5.02	6.67	4.66	4.20	0.25 (0.01)	0.14 (0.00)	0.39 (0.01)	8
Leoni 4	4.59	6.44	4.33	3.85	0.25 (0.01)	0.27 (0.01)	0.52 (0.01)	automatical (default 4)
Sophie	4.55	6.44	4.33	3.91	0.06 (0.01)	0.11 (0.01)	0.17 (0.01)	automatical
Servo-n	4.57	6.03	4.40	3.86	0.06 (0.01)	0.12 (0.01)	0.18 (0.01)	8
Babylog	4.34	6.24	4.59	4.09	0.27 (0.01)	0.24 (0.01)	0.51 (0.01)	6
F120	4.85	6.81	4.51	4.03	0.28 (0.03)	0.30 (0.03)	0.58 (0.02)	12
Perivent	3.69	6.37	4.37	3.89	0.38 (0.02)	0.41 (0.01)	0.79 (0.02)	8
Servo-i	2.32	5.23	no quantitative analysis useful					
Bubble	3.64	7.63	3.49*	3.87*	0.04 (0.01) *	0.04 (0.01) *	0.08 (0.01) *	variable
Hamilton-T1	4.63	5.68	4.42	4.08	0.04 (0.01)	0.04 (0.01)	0.08 (0.02)	automatical

B

CPAP-Device	P_{min} $[cmH_2O]$	P_{max} $[cmH_2O]$	$V_{tid}ex$ [ml]	$V_{tid}in$ [ml]	$iWOB_{ex}$ [mJ/breath] (SD)	$iWOB_{in}$ [mJ/breath] (SD)	$iWOB_{ges}$ [mJ/breath] (SD)	Flow [ml/min]
Benveniste	4.60	5.76	13.93	13.18	0.31 (0.05)	0.33 (0.04)	0.64 (0.06)	variable
NeoBreathe	4.68	5.58	13.99	13.26	0.15 (0.04)	0.24 (0.04)	0.38 (0.06)	8
InfantFlow	4.09	5.81	14.05	13.13	0.56 (0.07)	0.82 (0.05)	1.39 (0.10)	variable
RPAP	4.34	5.79	13.88	13.08	0.68 (0.05)	0.58 (0.06)	1.26 (0.09)	12
Medijet	No measurement possible since CPAP not adjustable							
Eve	4.57	6.40	14.19	13.38	0.56 (0.02)	0.33 (0.01)	0.89 (0.03)	automatical
Leoni +	4.65	8.25	14.52	13.43	1.79 (0.05)	0.76 (0.01)	2.55 (0.05)	8
Leoni 4	2.99	7.55	14.15	12.87	2.19 (0.11)	2.00 (0.07)	4.19 (0.08)	automatical (default 4)
Sophie	4.21	8.16	14.02	13.05	0.79 (0.11)	0.52 (0.08)	1.31 (0.07)	automatical
Servo-n	4.13	7.34	14.09	13.29	0.79 (0.02)	0.48 (0.01)	1.28 (0.02)	8
Babylog	3.59	7.99	13.92	12.79	2.13 (0.02)	1.42 (0.02)	3.55 (0.03)	6
F120	4.93	10.15	14.21	12.97	2.41 (0.19)	1.88 (0.08)	4.29 (0.18)	12
Perivent	2.26	8.94	14.08	12.79	3.80 (0.06)	2.92 (0.04)	6.72 (0.06)	8
Servo-i	3.57	12.62	no quantitative analysis useful					
Bubble	3.42	7.90	13.47*	13.13*	0.26 (0.04) *	0.17 (0.04) *	0.43 (0.05) *	variable
Hamilton-T1	4.30	6.73	4.53	4.15	0.05 (0.02)	0.06 (0.02)	0.10 (0.03)	automatical

A: simulated preterm; **B:** simulated term newborn; * indicates the values based on the smoothed curve of the bubble CPAP with rolled mean calculation

Table S4: Result of the measurements CPAP 10 cmH_2O

A

CPAP-Device	P_{min} [cmH_2O]	P_{max} [cmH_2O]	$V_{tid}ex$ [ml]	$V_{tid}in$ [ml]	$iWOB_{ex}$ [mJ/breath] (SD)	$iWOB_{in}$ [mJ/breath] (SD)	$iWOB_{ges}$ [mJ/breath] (SD)	Flow [l/min]
Benveniste	9.59	10.61	4.48	4.21	0.07 (0.02)	0.07 (0.02)	0.14 (0.03)	variable
NeoBreathe	9.63	10.56	4.47	4.18	0.06 (0.02)	0.07 (0.01)	0.13 (0.03)	13
InfantFlow	9.33	10.67	4.55	4.37	0.09 (0.03)	0.11 (0.03)	0.20 (0.04)	variable
RPAP	9.66	10.71	4.47	4.17	0.07 (0.08)	0.12 (0.03)	0.19 (0.10)	12
Medijet	8.35	11.47	4.54	3.93	0.44 (0.01)	0.47 (0.01)	0.91 (0.01)	13
Eve	9.70	10.60	4.58	4.24	0.08 (0.01)	0.07 (0.00)	0.15 (0.01)	automatical
Leoni +	9.95	11.47	4.64	4.19	0.19 (0.01)	0.14 (0.01)	0.33 (0.01)	8
Leoni 4	9	12.72	4.56	4.04	0.28 (0.01)	0.32 (0.01)	0.60 (0.01)	automatical (default 4)
Sophie	9.45	10.84	4.69	4.28	0.09 (0.01)	0.08 (0.01)	0.17 (0.01)	automatical
Servo-n	9.53	11.18	4.52	3.97	0.09 (0.00)	0.12 (0.00)	0.20 (0.01)	8
Babylog	9.05	10.96	4.61	4.12	0.27 (0.01)	0.23 (0.01)	0.50 (0.01)	6
F120	9.95	12.48	4.44	3.94	0.36 (0.06)	0.36 (0.05)	0.72 (0.03)	12
Perivent	7.45	11.86	4.59	4.03	0.63 (0.02)	0.80 (0.01)	1.42 (0.02)	8
Servo-i	6.34	10.23	no quantitative analysis useful					
Bubble	7.72	13.46	3.79*	4.22*	0.04 (0.01) *	0.03 (0.01) *	0.07 (0.02) *	variable
Hamilton-T1	9.52	10.87	14.18	13.46	0.18 (0.11)	0.15 (0.04)	0.32 (0.13)	automatical

B

CPAP-Device	P_{min} [cmH_2O]	P_{max} [cmH_2O]	$V_{tid}ex$ [ml]	$V_{tid}in$ [ml]	$iWOB_{ex}$ [mJ/breath] (SD)	$iWOB_{in}$ [mJ/breath] (SD)	$iWOB_{ges}$ [mJ/breath] (SD)	Flow [l/min]
Benveniste	9.39	10.85	13.86	12.94	0.19 (0.08)	0.33 (0.05)	0.52 (0.10)	variable
NeoBreathe	9.50	10.79	14.13	13.39	0.15 (0.07)	0.27 (0.06)	0.43 (0.10)	13
InfantFlow	8.65	11.76	13.92	12.87	0.75 (0.09)	1.05 (0.11)	1.80 (0.16)	variable
RPAP	9.13	11.35	13.83	12.91	0.96 (0.12)	0.83 (0.06)	1.80 (0.16)	12
Medijet	No measurement possible since CPAP not adjustable							
Eve	9.57	11.30	14.04	13.24	0.52 (0.02)	0.32 (0.03)	0.84 (0.04)	automatical
Leoni +	9.62	12.66	14.33	13.35	1.47 (0.03)	0.72 (0.01)	2.19 (0.03)	8
Leoni 4	8.26	12.65	14.12	12.91	2.09 (0.05)	1.72 (0.03)	3.82 (0.06)	automatical (default 4)
Sophie	9.14	12.56	14.57	13.56	0.77 (0.07)	0.45 (0.06)	1.22 (0.05)	automatical
Servo-n	9.17	12.53	14.00	12.90	0.65 (0.03)	0.42 (0.01)	1.07 (0.03)	8
Babylog	8.59	12.74	13.85	12.69	2.03 (0.04)	1.35 (0.02)	3.38 (0.05)	6
F120	8.96	13.20	14.12	12.78	2.22 (0.41)	1.48 (0.32)	3.69 (0.24)	12
Perivent	4.63	15.24	14.14	12.56	4.95 (0.23)	5.41 (0.31)	10.36 (0.38)	8
Servo-i	8.47	19.03	no quantitative analysis useful					
Bubble	7.24	14.82	13.43*	12.96*	0.25 (0.05) *	0.17 (0.04) *	0.42 (0.05) *	variable
Hamilton-T1	9.17	11.90	14.21	13.40	0.05 (0.07)	0.27 (0.08)	0.32 (0.11)	automatical

A: simulated preterm; **B:** simulated term newborn; * indicates the values based on the smoothed curve of the bubble CPAP with rolled mean calculation

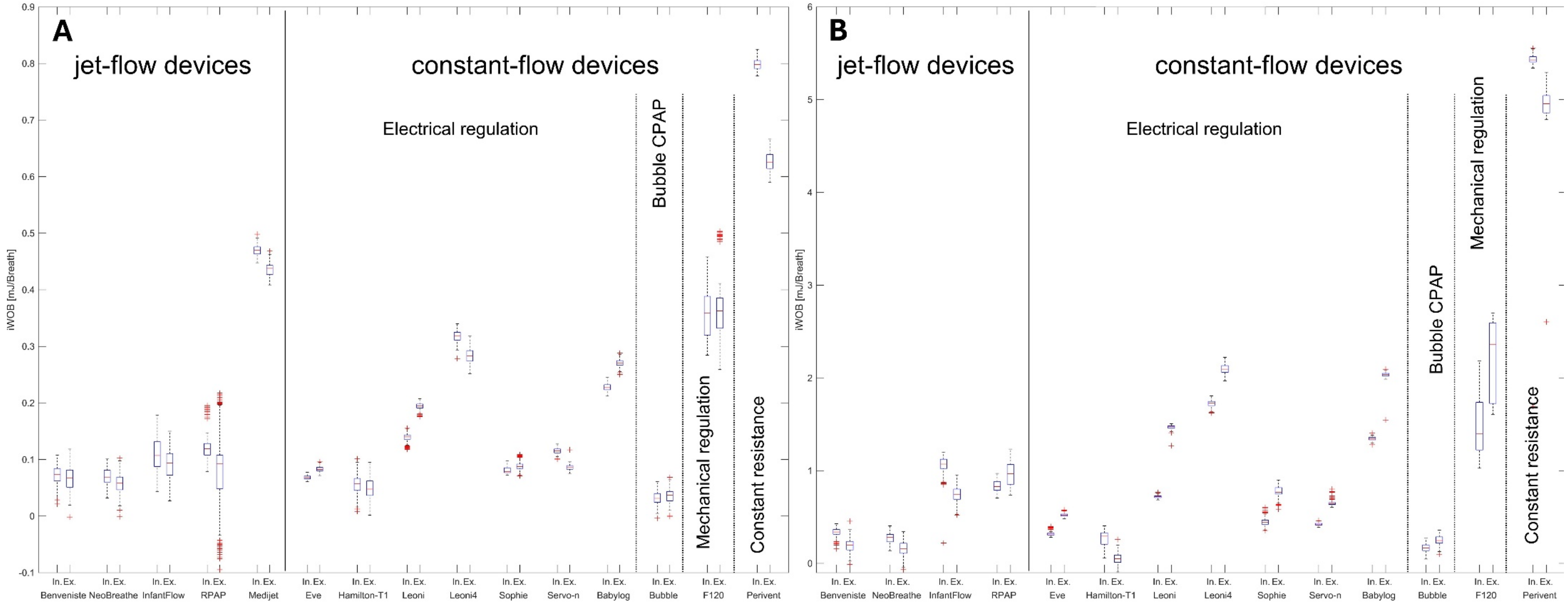

Figure S2: Box-plots of proportion of additional iWOB$_{insp}$ (CPAP 10 cmH$_2$O): Inspiratory iWOB in a simulated 1000 g preterm (A) resp. 3000 g term (B) model (CPAP 10 cmH2O, see table S3 for additional parameters); no iWOB determination feasible in term model for Medijet® and both models for Servo-i®

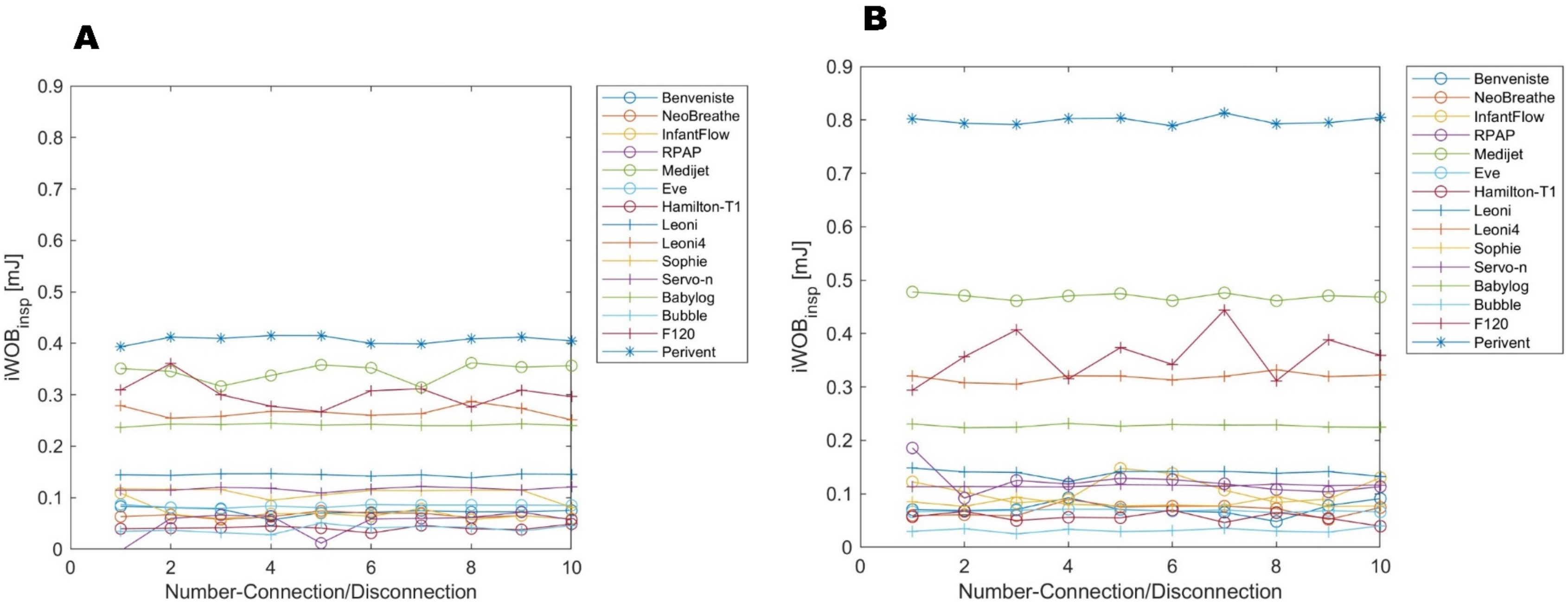

Figure S3: Interaction plot of inspiratory iWOB in the simulated preterm and A: CPAP 5 or B: CPAP 10 cmH_2O

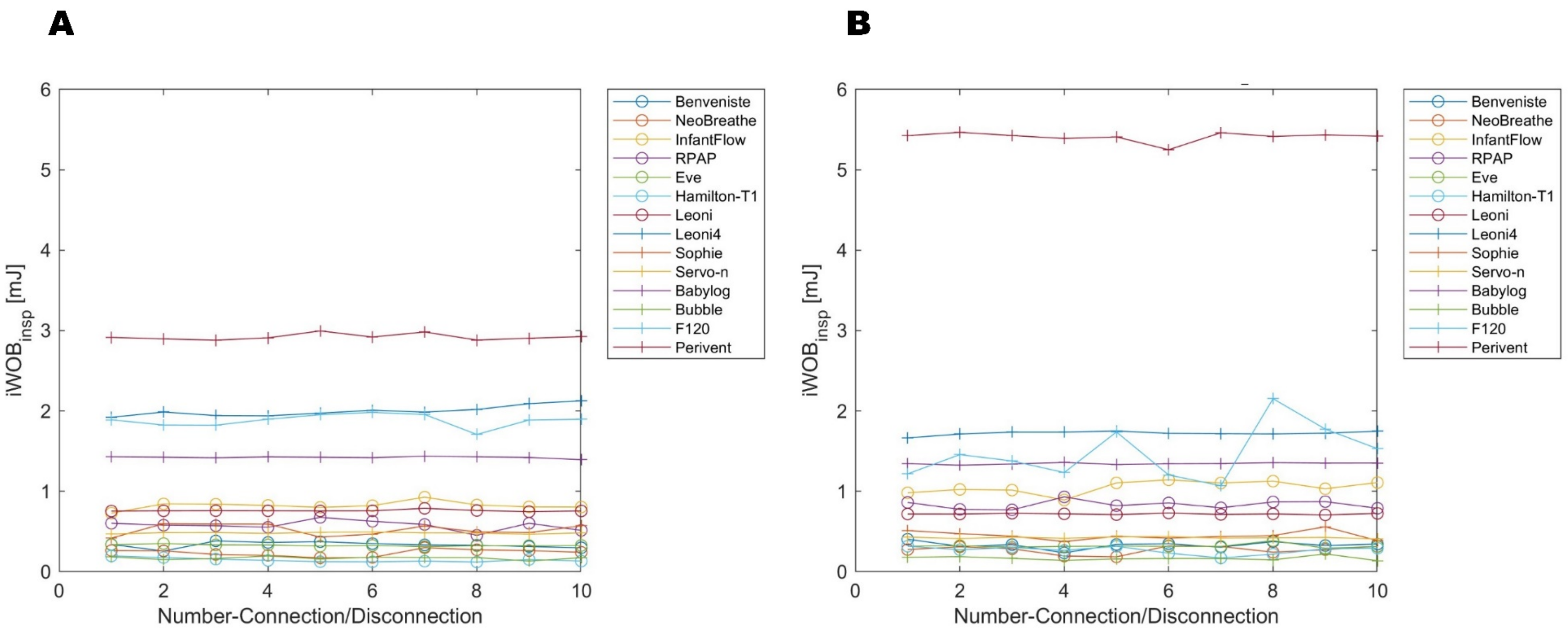

Figure S4: Interaction plot of inspiratory iWOB in the simulated term and A: CPAP 5 or B: CPAP 10 cmH_2O

Online Supplemental References

S1. Abbasi S, Bhutani VK. Pulmonary mechanics and energetics of normal, non-ventilated low birthweight infants. *Pediatr Pulmonol*. 1990 1990;8(2):89-95. doi:10.1002/ppul.1950080206

S2. Cook CD, Cherry RB, O'Brien D, Karlberg P, Smith CA. Studies of respiratory physiology in the newborn infant. I. Observations on normal premature and full-term infants. *J Clin Invest*. Jul 1955;34(7, Part 1):975-82. doi:10.1172/JCI103165

S3. Cook CD, Sutherland JM, Segal S, et al. Studies of Respiratory Physiology in the Newborn Infant. III. Measurements of Mechanics of Respiration1. *Journal of Clinical Investigation*. 1957/03// 1957;36(3):440-448.

S4. Estol P, Piriz H, Pintos L, Nieto F, Simini F. Assessment of pulmonary dynamics in normal newborns: a pneumotachographic method. *J Perinat Med*. 1988 1988;16(3):183-92. doi:10.1515/jpme.1988.16.3.183
